# Why Do Employees Show Pro-Environmental Behaviors? A Perspective of Environment Social Responsibility

**DOI:** 10.3390/bs13060463

**Published:** 2023-06-02

**Authors:** Shih-Chin Lee, Stanley Y. B. Huang, Ling Hu, Tai-Wei Chang

**Affiliations:** 1 Department of Finance, Chihlee University of Technology, New Taipei City 22050, Taiwan; 2 Master Program of Financial Technology, Ming Chuan University, Taipei City 111, Taiwan; 3 Department of Finance, Hsing Wu University, New Taipei City 244, Taiwan; 4 Graduate School of Resources Management and Decision Science, National Defense University, Taipei City 112, Taiwan

**Keywords:** green commitment, environment social responsibility, institutional pressure, pro-environmental behaviors, sustainability

## Abstract

This research uses social identity theory to propose that environmental social responsibility perceptions influence green commitment, and then influence pro-environmental behaviors, which are moderated by institutional pressure. Data were collected from 100 employees of technology firms in Taiwan, and the results support all hypotheses. This research chose technology firms as empirical data because Taiwan’s technological level is known to the world, which can reduce sampling errors caused by the lack of environmental knowledge. Finally, this research not only advances the literature on sustainability issues in organizational management but also provides a paradigm to help firms implement pro-environmental behaviors to achieve competitive advantage and sustainable development goals.

## 1. Introduction

To obtain a green competitive advantage [1,2,3], contemporary firms must inspire employees to show pro-environmental behavior, as environmental behaviors have been confirmed as one kind of source for sustainable development [4,5,6]. Pro-environmental behaviors denote people’s environmental behaviors toward sustainability [7,8,9]. However, previous studies have neglected how environmental social responsibility leads to pro-environmental behaviors; thus, firms have no idea how to formulate strategies that can induce employees’ pro-environmental behaviors [10,11,12].

This research adopts social identity theory [13,14,15] to put forward a moderated mediation model, and that model describes how environmental social responsibility affects pro-environmental behaviors through the mediating role of green commitment and the moderating role of institutional pressure, which is the first contribution of this research. Examining the key antecedents of pro-environmental behaviors is important because it can help firms create an effective strategy to drive employees’ pro-environmental behaviors, which will achieve long-term sustainable management goals [16,17,18]. Therefore, this research adopts the social identity theory [13,14,15] to propose that environmental social responsibility is an important driver of pro-environmental behaviors through the mediating role of green commitment because an employee wants to show work behaviors that meet his/her work value. When the employee continuously works in the firm, his/her work value must meet the firm’s value. In addition, environmental social responsibility adoption represents that the firm’s value is toward environmental performance, and it will shape the employee’s environmental value to form a green commitment based on social identity theory. The green commitment may affect pro-environmental behaviors, as the employee intends to show behaviors (pro-environmental behaviors) that are in line with his/her value (green commitment) based on social identity theory. Finally, this research uses institutional pressure as a boundary condition variable, because the higher the level of institutional pressure on the employee, the stronger the willingness of the employee to behave pro-environmentally due to his/her green commitment. In short, the main goal of this research is to open the black box for pro-environmental behaviors with its antecedents based on social identity theory, which is the second contribution of this research.

This research pays attention to the concept of “growths” for green commitment perception, pro-environmental behaviors, and institutional pressure perception, as there is an important flaw in behavioral science research [19,20,21]. That said, past empirical research for behavioral science uses cross-sectional designs to infer longitudinal causality between constructs so the assumption of behavioral science that people should modify their attitudes and behaviors based on how they translate environmental contexts has not been investigated in detail. The second goal of this research is to address the research gap in the behavioral science literature, and IBM SPSS software 25 [22] was adopted to analyze 100 employees of technology firms to fill the gap, which is the third contribution of this research.

**Figure 1:**
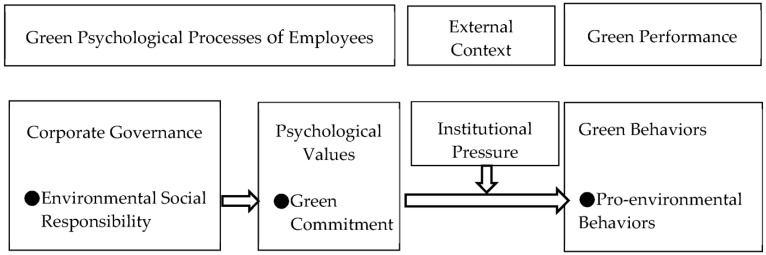
Comprehensive psychological model.

Finally, previous research pays little attention to how the green psychological processes of employees induce green performance; therefore, this research investigates the impact of environmental social responsibility through the mediating effect of green commitment and the moderating effect of pro-environmental behaviors. This research addresses a comprehensive psychological model to explore the mechanism between corporate governance, psychological values, external context, and green performance based on social identity theory (please see Figure 1).

## 2. Literature Review

**Figure 2:**
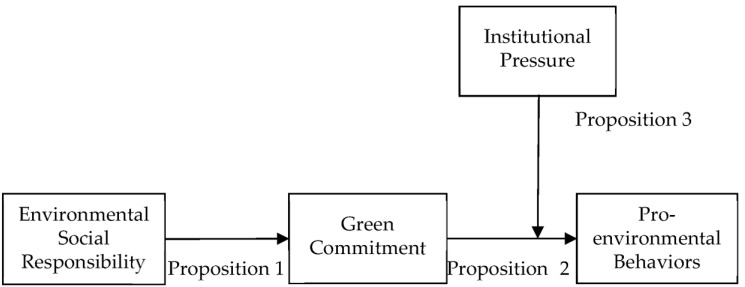
Theoretical model of this research.

This research poses the theoretical model in Figure 2.

Based on the above literature review, this research addresses the theoretical model in Figure 1. Figure 1 describes how environmental social responsibility induces green commitment and then induces pro-environmental behaviors, which are moderated by institutional pressure.

### 2.1. Environmental Social Responsibility and Green Commitment

Environmental social responsibility represents environmentally responsible practice policies that satisfy various stakeholders [23,24,25]. For example, firms transform narrow economic interests and lawful requirements into environmental concerns and combine these concerns into firms’ commercial activities, operations, and competitive strategies. These commercial activities, operations, and competitive strategies will form firms’ values toward environmental concerns. Based on social identity theory, employees who identify their firms’ values, will modify their work values toward the firms’ values, which will form a green commitment. However, if employees cannot modify their work value to meet their firms’ values, they will leave their firms [26,27,28,29], which will not form a green commitment.

Thus, environmental social responsibility will shape employees’ work values to form a green commitment, because employees who continue to work in the firm must identify with the firm’s values based on social identity theory. Past empirical studies also support this perspective and believe that the environmental values of organizations will shape the green commitment of employees [30,31]. In addition, a firm with a high reputation for environmental social responsibility will also attract new employees with the same value and, therefore, will attract employees with high environmental values into the company [32].

**Hypothesis 1.**  *Environmental* *social responsibility at the initial time causes greater growth of green commitment.*

### 2.2. Green Commitment and Pro-Environmental Behaviors

Past research has suggested that people exhibit pro-environmental behaviors because people intend to satisfy their self-values [33,34,35]. Thus, people with high levels of green commitment exhibit pro-environmental behaviors because they intend to exhibit behaviors that satisfy their green commitment (self-values). Based on social identity theory [13,14,15], employees internalize organizational values and goals as self-values (e.g., green commitment), and exhibit behaviors that are consistent with organizational values (e.g., pro-environmental behaviors) and goals. Past empirical studies also support this perspective and believe that environmental values will drive employees to show environmental behaviors [36,37]. Indeed, commitment has been proven as a key antecedent of pro-organizational behaviors [38,39].

**Hypothesis 2.**  *Greater growth of green commitment causes greater growth of pro-environmental behaviors*.

### 2.3. Institutional Pressure as a Moderator

Institutional pressure denotes the typical case of laws and government entities forcing a company toward external environmentalism [40,41,42]. Past studies have pointed out that institutional pressures will influence firms’ decisions because firms want to meet the expectations of stakeholders (e.g., the social public, social media, and government). Therefore, when firms face institutional pressures, these firms will modify their organizational values toward organizational environment performance. Simultaneously, the employees of these firms will also show similar environmental behaviors based on social identity theory [13,14,15], because these employees’ work values are in line with their organizational values.

This research believes that institutional pressure can boost the relationship between green commitment and pro-environmental behavior. When employees face greater institutional pressure, they are likely to exhibit more pro-environmental behavior caused by a green commitment to meet the expectation of stakeholders than employees facing lower institutional pressure. Past empirical studies also support this perspective and believe that institutional pressure will moderate the relationship between environment-related variables and their antecedents [43,44,45].

**Hypothesis 3.**  *Greater growth of institutional pressures moderates the relationship between greater growth of green commitment and greater growth of pro-environmental behaviors.*

## 3. Methodology

### 3.1. Measurements

This research uses Wei et al.’s [25] scale to measure environmental social responsibility—an example item is “our firms’ production process decreased more environmental pollution than our firms’ major competitors.” Green commitment is evaluated by Ren et al. [31]—an example item is “I feel a sense of duty to support the environmental efforts of my company.” Pro-environmental behaviors are evaluated by Lamm et al. [46]—an example item is “I feel that I am a person who properly disposes of electronic waste.” Finally, this research evaluates institutional pressures through Wu et al.’s [47] scale—an example item is “I think my firm’s green environmental management will be affected by the local government’s environmental regulations.” All items were evaluated by the 5-point Likert scale (please see Appendix A).

### 3.2. Data Collection

For this research, two information technology associations in Taiwan were contacted in order to obtain a sample list of 130 employees. A total of 230 employees were contacted, and 100 employees were willing to participate in the research survey. To increase response rates, participants were given USD 16.00 worth of gift vouchers.

Initially, an email was sent to the participants (employees) requesting that they complete the questionnaire about their environmental social responsibility perception, green commitment perception, pro-environmental behaviors, and institutional pressure perception. Subsequently, 100 responses were received, which represents a 100% response rate.

Next, a second email was sent requesting that the employees complete the second questionnaire about their green commitment perception, pro-environmental behaviors, and institutional pressure perception. Subsequently, 100 responses were received, which represents a 100% response rate.

Finally, a third email was sent requesting that the employees complete the third questionnaire about the same variables. Again, 100 responses were received, which represents a 100% response rate.

This research adopts the sampling design with the three-time points, as it can alleviate common methodological variance [48]. Among the 100 samples, 60% were male, the average age was 35 years old, 80% had at least a college degree, and the average working experience was three years (please see Table 1).

## 4. Analysis Results

### 4.1. Reliability and Validity Analysis

A confirmatory factors skill was adopted by this research to analyze the validity and reliability. Fornell and Lacker [49] stated that Cronbach’s alpha, composite reliability, and average variance extracted should be greater than 0.7, 0.6, and 0.5, respectively. The Cronbach’s alpha of the four constructs is all greater than 0.7. The composition reliability of the four constructs is all greater than 0.6. The average variance extracted from the four constructs is all greater than 0.5. Therefore, the validity and reliability of environmental social responsibility, green commitment, pro-environmental behaviors, and institutional pressures are valid (please see Table 2).

### 4.2. Analysis Results

This research demonstrated the result of the analysis in Table 3 and the IBM SPSS software 25 [22] was employed to analyze the relationships between these constructs. Environmental social responsibility at the initial time significantly influences greater growth of green commitment (Beta = 0.31, *p* < 0.01), which confirms Hypothesis 1. Hypothesis 1 proposes that greater environmental social responsibility perceptions of employees at the initial time will lead to employees showing greater green commitment.

Greater growth of green commitment significantly causes greater growth of pro-environmental behaviors (Beta = 0.29, *p* < 0.01), which confirms Hypothesis 2. Hypothesis 2 proposes that employees who show greater green commitment will cause them to show greater pro-environmental behaviors over time.

Finally, greater growth of institutional pressures significantly moderates the relationship between greater growth of green commitment and greater growth of pro-environmental behaviors (Beta = 0.32, *p* < 0.01), which confirms Hypothesis 3. Hypothesis 3 proposes that employees who perceive greater institutional pressures will cause them to show greater pro-environmental behaviors caused by greater green commitment over time.

## 5. Discussion

### 5.1. Contribution

First, this research significantly promotes the connection of environmental social responsibility, green commitment, institutional pressures, and pro-environmental behaviors, which opens the black box of pro-environmental behaviors and their antecedents. Per the analysis result, this research conceptualizes pro-environmental behaviors and their antecedents, and the analysis results confirm the causal relationship between environmental social responsibility, green commitment, institutional pressures, and pro-environmental behaviors. Indeed, these antecedents of pro-environmental behaviors can help firms develop policies that promote pro-environmental behaviors to achieve sustainable development. Limited studies have investigated the relationship between environmental social responsibility and environmental behaviors, and this mechanism has not been detected through the key mediating role of green commitment and the moderating role of institutional pressures. In addition, this research adopts longitudinal data to confirm the causality relationships between these variables when previous surveys almost adopt cross-section data to confirm correlations, which demonstrates the incremental contribution of this research.

This research puts forward the impact of environmental social responsibility on pro-environmental behaviors from the mediation perspective of green commitment and moderation perspective of institutional pressures, and it detects a novel black-box mechanism, which is called through previous studies [50,51,52]. This mechanism is important because technology firms can create effective strategies based on the theoretical framework of this research to drive pro-environmental behaviors. Pro-environmental behaviors have been confirmed as an important source of sustainable development; therefore, technology firms must make effective policies to drive employees’ pro-environmental behaviors.

Although past research has examined the positive impacts of environmental social responsibility [53,54,55], limited research investigates how the mediating perspective of green commitment and the moderating perspective of institutional pressure affect the domain of employee sustainable behavior. Consequently, past research ignores the key position of green commitment and institutional pressures for pro-environmental behaviors. Therefore, this research has a significant incremental contribution to the literature on social responsibility and environmental behaviors.

In practice, although a firm cannot adopt educational training to increase its environmental social responsibility adoption intention, the firm can still use educational training to increase all employees’ environmental knowledge. If all employees of the firm have a high level of environmental knowledge, the firm may prefer to adopt environmental social responsibility policies in line with employees’ work values, which can achieve the goal of sustainable management.

### 5.2. Future Research and Limitations

In the theoretical framework (Figure 1), there may be other variables that can replace environmental social responsibility, and further study should extract other key variables. Although this research adopts longitudinal data to analyze the theoretical model, the causality relationships should be confirmed through more investigations. In addition, although this research proposes the three hypotheses through the past literature and theory, there is little evidence to confirm the validity of the theoretical model. The “hypotheses” should be empirically confirmed for robustness through more data and should be tested for more antecedents. More analytical methods should be applied to verify the theoretical model of this research, such as neural networks, Hierarchical Linear Modeling, or qualitative research methods.

Next, this research surveyed 100 samples in Taiwan, and further study must survey different industries and countries to analyze the validity of generalization for the theoretical model in this research. Furthermore, this research is only a communication article; thus, future research needs to collect more references and more arguments for the three hypotheses.

Finally, this research adopts social identity theory to put forward the theoretical model, but other theories may meet the theoretical model, which is left for future research to explore these theories in different contexts. For example, Confucian cultures in Asia and Europe may be quite different, resulting in different results for samples from different cultures. In addition, the self-consistent theory [56] may be a possible theory to explain why employees demonstrate pro-environmental behaviors since these employees want to demonstrate environmental behaviors to meet their environmental responsibility value.

## 6. Conclusions

This research opens the black box for implementing pro-environmental behaviors and sets up a new milestone of sustainable development of technology firms. This research surveyed 100 employees of technology firms through three-time points in Taiwan to analyze the theoretical model. The analysis results support that environmental social responsibility indeed causes green commitment and pro-environmental behaviors, and that relationship is moderated by institutional pressure. The analysis results also fill the research gaps of pro-environmental behaviors. Expressly, technology firms can implement pro-environmental behaviors through environmental social responsibility adoption to achieve the goal of sustainable development. Finally, this research not only advances the literature on environmental behaviors from environmental social responsibility but also provides a paradigm to help firms implement pro-environmental behaviors to achieve competitive advantage and sustainable development goals.

## Figures and Tables

**Table 1:** Demographics.

Gender	Percentage
Male	60%
Female	40%
Age	Percentage
18~30	26%
31~40	49%
41~50	20%
51~60	5%
Education Level	Percentage
High School	10%
University Degree	80%
Master’s Degree or Above	10%
Working Experience	Percentage
2 years or less	11%
2~3 years	39%
3~4 years	41%
4 years or above	9%

**Table 2:** Validity and reliability analysis.

Constructs	Items	λ	Cronbach’s α	Composite Reliability	Average Variation Extracted
Environmental Social Responsibility	ESR01	0.76 **	0.87	0.89	0.63
	ESR02	0.82 **			
	ESR03	0.85 **			
	ESR04	0.75 **			
Green Commitment	GC01	0.76 **	0.92	0.91	0.61
	GC02	0.82 **			
	GC03	0.80 **			
	GC04	0.75 **			
	GC05	0.76 **			
	GC06	0.71 **			
	GC07	0.85 **			
Institutional Pressure	IP01	0.72 **	0.88	0.90	0.59
	IP02	0.83 **			
	IP03	0.84 **			
	IP04	0.73 **			
	IP05	0.71 **			
Pro-environmental Behaviors	PB01	0.72 **	0.94	0.93	0.58
	PB02	0.83 **			
	PB03	0.81 **			
	PB04	0.73 **			
	PB05	0.71 **			
	PB06	0.71 **			
	PB07	0.85 **			
	PB08	0.75 **			
	PB09	0.75 **			
	PB10	0.76 **			
	PB11	0.73 **			
	PB12	0.75 **			

Notes: ** *p* < 0.01; RMR = 0.048; RMSEA = 0.042; GFI= 0.92; CFI = 0.91; NFI = 0.93.

**Table 3:** Path analysis results.

Hypothesis	Relationship Path	Coefficient	R^2^
Hypothesis 1	Environmental Social Responsibility -> Green Commitment	0.31 **	0.37
Hypothesis 2	Green Commitment -> Pro-environmental Behaviors	0.29 **	0.42
Hypothesis 3	Institutional Pressures × Green Commitment -> Pro-environmental Behaviors	0.32 **	0.45

Note: ** *p* < 0.01.

## Appendix A

**Table A1:** The items of environmental social responsibility perception, green commitment perception, pro-environmental behaviors, and institutional pressure.

Variable	Evaluation Items
Environmental Social Responsibility [25]	1. Our products are more environmentally friendly than our major competitors. 2. Our production process requires fewer natural resources than our major competitors. 3. Our production process decreases environmental pollution more than our major competitors. 4. Our products are easier to recycle for reuse than our major competitors.
Green Commitment [31]	1. I would feel guilty about not supporting the environmental efforts of my company. 2. The environmental concern of my company means a lot to me. 3. I feel a sense of duty to support the environmental efforts of my company. 4. I feel as if my company’s environmental problems are my own. 5. I feel personally attached to the environmental concern of my company. 6. I feel an obligation to support the environmental efforts of my company 7. I strongly value the environmental efforts of my company
Institutional Pressures [47]	1. The green environmental management of our firm will be influenced by the central government’s environmental regulations. 2. The green environmental management of our firm will be influenced by the regional government’s environmental regulations. 3. Potential conflicts between products and environmental regulations will affect our firm’s green environmental management. 4. The green environmental management of our firm will be influenced by buyers’ environmental regulations. 5. The green environmental management of our firm will be influenced by the cost of pollution prevention.
Pro-environmental Behaviors [46]	1. I am a person who recycles my bottles, cans, and other containers. 2. I am a person who uses scrap paper for notes instead of fresh paper. 3. I am a person who prints double-sided. 4. I am a person who turns off my lights when leaving my office for any reason. 5. I am a person who recycles used paper. 6. I am a person who powers off my computer when away for more than 3 h. 7. I am a person who turns off the lights in a vacant room. 8. I am a person who powers down all desk electronics at the end of the day. 9. I am a person who uses a reusable water bottle instead of a paper cup at the water cooler or faucet. 10. I am a person who uses a reusable coffee cup instead of a paper cup. 11. I am a person who properly disposes of electronic waste. 12. I am a person who makes sure all the lights are turned off if I am the last to leave.
